# Distinguishing Among Evolutionary Forces Acting on Genome-Wide Base Composition: Computer Simulation Analysis of Approximate Methods for Inferring Site Frequency Spectra of Derived Mutations

**DOI:** 10.1534/g3.117.300512

**Published:** 2018-03-27

**Authors:** Tomotaka Matsumoto, Hiroshi Akashi

**Affiliations:** * Division of Evolutionary Genetics, National Institute of Genetics, Mishima, Shizuoka, Japan; † Department of Genetics, The Graduate University for Advanced Studies (SOKENDAI), Mishima, Shizuoka, Japan

**Keywords:** ancestral reconstruction, codon usage, GC content, unfolded site frequency spectrum, nucleotide substitution

## Abstract

Inferred ancestral nucleotide states are increasingly employed in analyses of within- and between -species genome variation. Although numerous studies have focused on ancestral inference among distantly related lineages, approaches to infer ancestral states in polymorphism data have received less attention. Recently developed approaches that employ complex transition matrices allow us to infer ancestral nucleotide sequence in various evolutionary scenarios of base composition. However, the requirement of a single gene tree to calculate a likelihood is an important limitation for conducting ancestral inference using within-species variation in recombining genomes. To resolve this problem, and to extend the applicability of ancestral inference in studies of base composition evolution, we first evaluate three previously proposed methods to infer ancestral nucleotide sequences among within- and between-species sequence variation data. The methods employ a single allele, bifurcating tree, or a star tree for within-species variation data. Using simulated nucleotide sequences, we employ ancestral inference to infer fixations and polymorphisms. We find that all three methods show biased inference. We modify the bifurcating tree method to include weights to adjust for an expected site frequency spectrum, “bifurcating tree with weighting” (BTW). Our simulation analysis show that the BTW method can substantially improve the reliability and robustness of ancestral inference in a range of scenarios that include non-neutral and/or non-stationary base composition evolution.

Knowledge of ancestral sequences allows inference of changes on each branch of a phylogenetic tree and can be highly useful for evolutionary analyses. In some cases, ancestral DNA samples can be sequenced directly, but for the vast majority of studies, ancestral states must be inferred statistically. Several methods have been developed to infer states at ancestral nodes based on likelihood calculation assuming a phylogenetic tree (*e.g.*, Yang *et al.* 1995; Koshi and Goldstein 1996; Pagel *et al.* 2004; Yang 2007; Matsumoto *et al.* 2015). These methods can incorporate complex transition rate matrices and allow accurate inference of ancestral states for nucleotide and amino acid sequences in a range of evolutionary scenarios. However, transition rate matrix/fixed tree (TRMFT) approaches require knowledge of phylogenetic relationships among genome sequences and assume that the same relationships hold across sites (Yang 2006). This restriction is problematic for ancestral inference for sequences taken from a single population because recombination can cause different genomic regions to have different genealogies.

Determining patterns and testing the causes of base composition evolution often relies on ancestral inference (*e.g.*, Akashi 1995; Kliman 1999; Begun 2001; Takano-Shimizu 2001; Duret *et al.* 2002; Gaucher *et al.* 2003, 2008; Groussin and Gouy 2011; Aoki *et al.* 2013; Terekhanova *et al.* 2013; Glémin *et al.* 2015, Bolívar *et al.* 2015; Clément *et al.* 2017). Previous studies have revealed fixation biases (weak selection and/or biased gene conversion) elevating GC content (*e.g.*, Akashi 1995, 1999; Kliman 1999; Maside *et al.* 2004; Galtier *et al.* 2006; Haddrill and Charlesworth 2008; Muyle *et al.* 2011; Lachance and Tishkoff 2014; Glémin *et al.* 2015) and have shown strong lineage-dependence in base composition evolution (*e.g.*, Akashi 1996; Duret *et al.* 2002; Powell *et al.* 2003; Akashi *et al.* 2006; Nielsen *et al.* 2006; Singh *et al.* 2009; Groussin and Gouy 2011; Lartillot 2012; Weber *et al.* 2014). Although long-term evolutionary analyses of base composition have incorporated sophisticated models using TRMFT ancestral inference methods, population genetic analyses of base composition evolution have employed a limited number of approximate methods for ancestral inference. Akashi (1995) and Akashi and Schaeffer (1997) inferred ancestral states of nucleotide sequences in *Drosophila* species using maximum parsimony (*i.e.*, assigning ancestral states to minimize the numbers of evolutionary changes required to explain the observed data). Clemente and Vogl (2012), Poh *et al.* (2012), Bolívar *et al.* (2015) and Clément *et al.* (2017) employed a similar approach but used only sites at which all outgroup species have the same state. In such cases, they assumed that the outgroup state is the ancestral condition in order to estimate the number of fixations and site frequency spectrum (SFS) of GC content-altering mutations.

Other studies have employed likelihood methods for ancestral inference assuming a common genealogy for all sites. Kern and Begun (2005) assigned a star tree to samples from a single species. In contrast, Akashi *et al.* (2006) and Matsumoto *et al.* (2016) assigned nucleotide states segregating within species to bifurcating lineages within each species. Finally, Jackson *et al.* (2017) employed single sequences from each sampled species to infer ancestral states and assumed identity of ancestral states at within and between species nodes. These approaches allow TRMFT ancestral inference with complex models (*e.g.*, different transition probabilities among 12 nucleotide mutation classes).

Assuming a single gene tree for polymorphism data is unrealistic for recombining regions and the reliability of the ancestral inference approaches described above for within- and among-species data are largely unknown (but see Hernandez *et al.* (2007) for maximum parsimony approach). Some studies have proposed Bayesian methods to consider phylogenetic uncertainty giving a prior distribution of phylogeny (Huelsenbeck and Bollback 2001, Pagel *et al.* 2004). However, these methods were developed to address uncertainty in deep phylogenies and their applicability to within species variation is unclear (the number of possible genealogies for a given region can be very large and trees can differ among regions). Previously, we implemented the GTR-NH_b_ model which allows lineage-specific transition rates and appears to be a robust approach to inferring ancestral states under several evolutionary scenarios of base composition (Matsumoto *et al.* 2015). This method is currently only available in the BASEML software package which employs a TRMFT ancestral inference method (Yang 2007).

In this study, we focus on TRMFT likelihood-based ancestral inference methods to study base composition evolution within and among closely related species. We use BASEML for ancestral inference and examine the accuracy of estimations of the numbers of fixations, polymorphic mutations and SFS using the methods that assign a single tree to within species polymorphism. We evaluated the accuracy of the three methods explained above. For simplicity, we refer to the methods in Jackson *et al.* (2017), Matsumoto *et al.* (2016) and Kern and Begun (2005) as “single allele” (SA), “bifurcating tree (BT)” and “star tree” (ST), respectively. We also introduce a new approach that incorporates allele frequency information in the inference of ancestral states at polymorphic sites (“bifurcating tree with weighting (BTW)”)

In order to access the reliability of the methods, we generated nucleotide sequences by computer simulation assuming neutral or nearly-neutral base composition evolution. Using nucleotide sequences generated for six species, we employed the tree assignment methods described above and conducted ancestral inference under a likelihood based method assuming a complex nucleotide substitution model, GTR-NH_b_. For these data, SA, BT, and ST approaches suffer from considerable estimation bias. However, the BTW method using the expected SFS under neutral equilibrium (BTW_ne_) allows accurate estimation of the SFS in several scenarios of base composition evolution and this accuracy is relatively robust to the fit of the expected SFS. In addition, even when the actual SFS shows strong departure from the SFS under neutral equilibrium, an iterative approach to BTW substantially increases the accuracy of the estimation. Our analyses support that ancestral inference using the approximate BTW method may be sufficiently reliable for within-population analyses of the base composition of non-coding regions, introns or codon usage bias in a wide range of evolutionary scenarios. However, we point out some limitations of models that do not incorporate population genetic processes, especially ancestral polymorphism.

## Methods

We describe below (1) the computer simulation to generate nucleotide sequences, (2) the process of inferring polymorphisms and fixations from ancestral inference results, and (3) the four ancestral inference methods evaluated in this study. Acronyms used in the text are summarized in Table 1.

**Table 1:** Key acronyms used in the text

Acronym	Meaning
SFS	site frequency spectrum
TRMFT	transition rate matrix/fixed tree likelihood-based method for sequence evolution inference
PFAP	parallel fixation of ancestral polymorphism
SA	single allele method for ancestral inference
BT	bifurcating tree method for ancestral inference
ST	star tree method for ancestral inference
BTW	bifurcating tree with weighting by expected SFS method for ancestral inference
SFS_ne_	site frequency spectrum under neutral equilibrium
BTW_ne_	BTW with weighting by expected SFS under neutral equilibrium
iterative BTW_est_	iterative BTW analysis with weighting by estimated SFS in the previous round
AWP	Averaging Weighted by Probability method for fixation counting
EMC	Expected Markov Counting method for fixation counting
*r*_pd_	ratio of the number of polymorphic sites to the number of fixed differences

### Computer simulation

**Figure 1:**
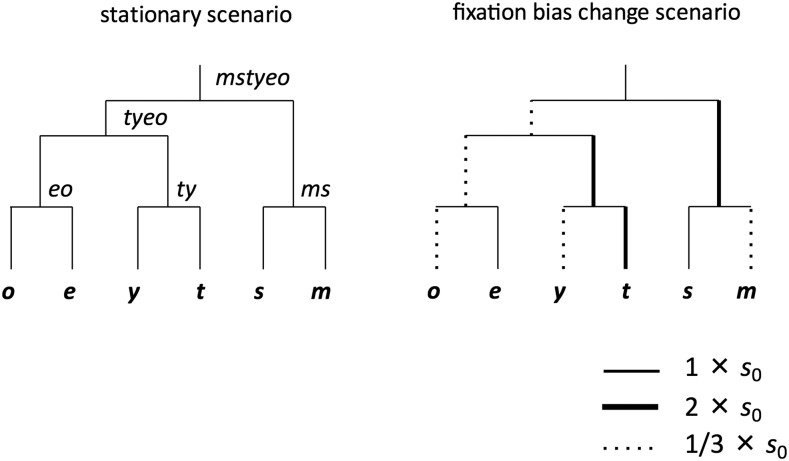
Phylogenetic relationships and evolutionary scenarios used to generate simulated sequences. Tree is a simplified depiction of relationships among six *D. melanogaster* subgroup species (Ko *et al.* 2003). Two selection schemes, stationary and fixation bias change scenarios were considered in the simulations. Lineage-specific selection intensities are expressed by different line-formatting in the phylogeny.

Computer simulations were designed to emulate base composition evolution among six species using a tree and approximate distances for the *Drosophila melanogaster* subgroup (Figure 1). In the discussion below, “*a-b* lineage” will refer to the lineage connecting nodes *a* and *b* and “*a* node (population)” will refer to node (or population) *a* using the node names in Figure 1. Forward-running simulations were conducted similarly to Matsumoto *et al.* (2016) except we consider a four- (rather than two-) state model of base composition. Each simulation was initiated with a “burn-in” process to allow populations to reach equilibrium base composition and a stationary frequency distribution for polymorphic sites. Burn-in parameters were set to: population size *N* = 1,000, mutation rates between any two nucleotides *u* = 2.5×10^−6^, and recombination rate between neighboring sites in each mating pair *r* = 0.01. Recombination rates of *Nr* = 10 give results close to free recombination expectations (Figure S1 in Matsumoto *et al.* 2016). We modeled haploid genomes with 10^5^ nucleotide sites. Evolution proceeds in discrete generations and in some scenarios, we assigned higher viability to individuals with elevated GC content with fitness of individual *j* having *x* A or T nucleotides calculated as *f_j_* = (1 - *s*)*^x^*. After viability selection, sequences undergo mutation and random pairing/recombination and the process is repeated with the resulting sequences. We will refer to selection coefficients and GC contents at the *mstyeo* node (end of burn-in) as *s*_0_ and GC_0_, respectively. Three values of GC_0_ were employed: 0.5 (*s*_0_ = 0), 0.7 (*s*_0_ = 0.00043) and 0.9 (*s*_0_ = 0.0011). Note that evolutionary dynamics under this scenario of weak selection favoring GC are indistinguishable from expectations under Nagylaki’s (1983) model of GC-biased gene conversion without selection. We will employ the term “fixation bias” to include selection and/or biased gene conversion favoring GC.

The initial base composition of all sequences was set to expected equilibrium values at the start of each burn-in and lineages were evolved for 20,000 generations. The resulting populations were used in downstream simulations. When a simulation reached time points for lineage splitting (internal *ms*, *ty*, *eo*, *tyeo*, *mstyeo*, Figure 1A), the population of the ancestral lineage was duplicated to create initial populations for the two descendent populations (ancestral polymorphism can segregate in the derived lineages but there is no gene flow after the split). We recorded sequence changes throughout the simulation to track actual numbers of fixations and polymorphic mutations, and SFS. 10 sequences were randomly chosen from each population shown in Figure 1 to test inference methods. Some of the analyses employed subsets or alterations of the samples as explained below.

The two main evolution scenarios considered in this study are shown in Figure 1. The first is a stationary process in which all lineages share burn-in parameter values. Lineage lengths were set to give rough correspondence to synonymous divergence within the *D. melanogaster* subgroup (Akashi *et al.* 2007): 3,610 generations for *mstyeo-tyeo* lineage, 10,840 generations for *mstyeo-ms* lineage, and 7,230 generations for all other lineages give expected sequence divergences of approximately 2.5%, 7.5% and 5%, respectively, in the stationary scenario with GC_0_ = 0.7. In our simulations, the expected time to the within-population most recent common ancestor (MRCA) is 2*N* = 2000 generations (for stationary, neutral evolution). Lineage lengths in these simulations are much greater than the expected time to MRCA and shared ancestral polymorphism should be rare among terminal node populations. The second scenario models non-stationary evolution, in which the *mstyeo-ms*, *tyeo-ty* and *ty-t* lineages are affected by stronger selection (2*s*_0_) and *ms-m*, *ty-y*, *eo-o* and *mstyeo-tyeo* lineages are affected by weaker selection (*s*_0_/3) compared with the burn-in values (Figure 1B). Other parameter values are identical to those in the stationary scenario. We refer to these non-stationary simulations as “fixation bias change” scenarios.

We also simulated non-stationary evolution with demographic changes. Two scenarios consider population size reductions in the *ms-m* lineage (demA: population size reduced to 100 at 7220^th^ generation, demB: population size reduced to 100 at 7130^th^ generation). The other two scenarios experience population size expansions in the *ms-m* lineage (demE: population size increased to 10000 at 7130^th^ generation and demF: population size increased to 10000 at 6230^th^ generation). The SFSs in the *m* population of these scenarios showed large deviation from the SFS under neutral equilibrium. Parameters other than population size were identical to those used in the stationary scenario for GC_0_ = 0.7. The dem scenarios are similar to the depiction in Figure 1A except for changes in *N* on the *ms-m* lineage. In this study, we focused on whether the deviations from the SFS under neutral equilibrium caused by the demographic change scenarios affect the accuracy of ancestral inference. We considered other demographic scenarios that have smaller impacts on the SFS (see Figure S7 and Table S2).

We conducted a computer simulation for each parameter set to produce data sets to test ancestral inference methods. Ancestral inference employed the species phylogeny shown in Figure 1 and we inferred probabilities of ancestral states using BASEML under the GTR-NH_b_ model (except where noted otherwise). For a given simulation, we replicated sampling and ancestral inference 100 times (all steps in the ancestral inference process were conducted independently for each replicate) to estimate confidence intervals on counts of mutations in three categories, *pu* (preferred to unpreferred, GC→AT, G or C to A or T), *up* (AT → GC) and *pp+uu* (GC → GC and AT → AT). “Polymorphic” or “segregating” sites will refer to sites at which multiple states are found within a population sample. “Fixations” will refer to derived states that are shared among the sampled alleles (including mutations common to the entire population as well as those shared within the sample but not the entire population). Fixations include mutations that arose after the split with a sister species as well as mutations that pre-date species splitting (ancestral polymorphism). For example, among fixations on the *ms-m* lineage, the majority originated after the *ms* node but a substantial fraction originated prior to this ancestral node (in the stationary scenario with GC_0_ = 0.5, this fraction was about 28%).

### Reconstructing polymorphisms and fixations

We conducted ancestral inference at internal nodes using the sequence samples and trees as input. Unless noted otherwise, all analyses employed the GTR-NH_b_ transition rate model implemented in BASEML software (Yang 2007; Matsumoto *et al.* 2015) and ancestral reconstructions were used to infer polymorphisms and fixations. Following Matsumoto *et al.* (2015), we ran BASEML 10 times and employed only the result with the highest likelihood. For inference of within-species polymorphisms, we compared estimated joint probabilities of ancestral states (probability for a set of ancestral states at all internal nodes) by BASEML and states of the 10 sequences sampled from the population. For example, consider a case where nine individuals have nucleotide A and one individual has nucleotide C at a given site among the sampled sequences. If the estimated ancestral states at this polymorphic site are A and C with probabilities 0.6 and 0.4, respectively, we counted 0.6 A → C polymorphic mutations in frequency class 1 (singletons) and 0.4 C → A polymorphic mutations in frequency class 9. Repeating this process across all sites, we estimated the number of polymorphic mutations and their SFS. Sites at which more than two nucleotides are segregating were filtered in all analyses; the proportion of such sites was < 1% among all sites and 5–7% among polymorphic sites.

Ancestral inference under GTR-NH_b_ allows estimation of the number of fixations considering lineage and mutation-category specific parameters. We examined the accuracy of the estimation of the number of fixations as follows. If the estimated states at ancestral node *a* and descendant node *b* are T and G with probabilities 0.6 and A and C with probability 0.4, respectively, we “counted” 0.6 T → G and 0.4 A → C fixations on the *a-b* lineage. This method, referred to as AWP (Averaging Weighted by Probability), slightly underestimates the numbers of fixations when multiple hits occur at a same site within a lineage (Matsumoto *et al.* 2015). In this study, we used AWP only for the BTW method and for other methods, we used EMC (Expected Markov Counting) implemented in BASEML in which the number of substitutions is counted over the Markov chain process of substitution considering the change of base composition along a branch (Matsumoto *et al.* 2015).

### Ancestral inference method 1: Single allele (SA)

We performed SA analyses using a single sequence sampled from each population as described in Jackson *et al.* (2017). First, we randomly sampled 10 sequences from each population in Figure 1 and then randomly selected one sequence for the ancestral inference step. We assumed that the states at the *ms*, *ty* and *eo* nodes are the ancestral states at polymorphic sites in the (*m* and *s*), (*t* and *y*), and (*e* and *o*) populations respectively (*i.e.*, in the *ms* clade, we assumed no fixations after the split between the *ms-m* and *ms-s* lineages). We estimated the numbers of polymorphic mutations and their SFS in the *a* population by comparing the inferred ancestral states at the *ab* node and the observed states in the sample of 10 sequences from the *a* population as explained above. We estimated the numbers of fixations on the *ab-a* lineage using the EMC approach.

### Ancestral inference method 2: Bifurcating tree (BT)

**Figure 2:**
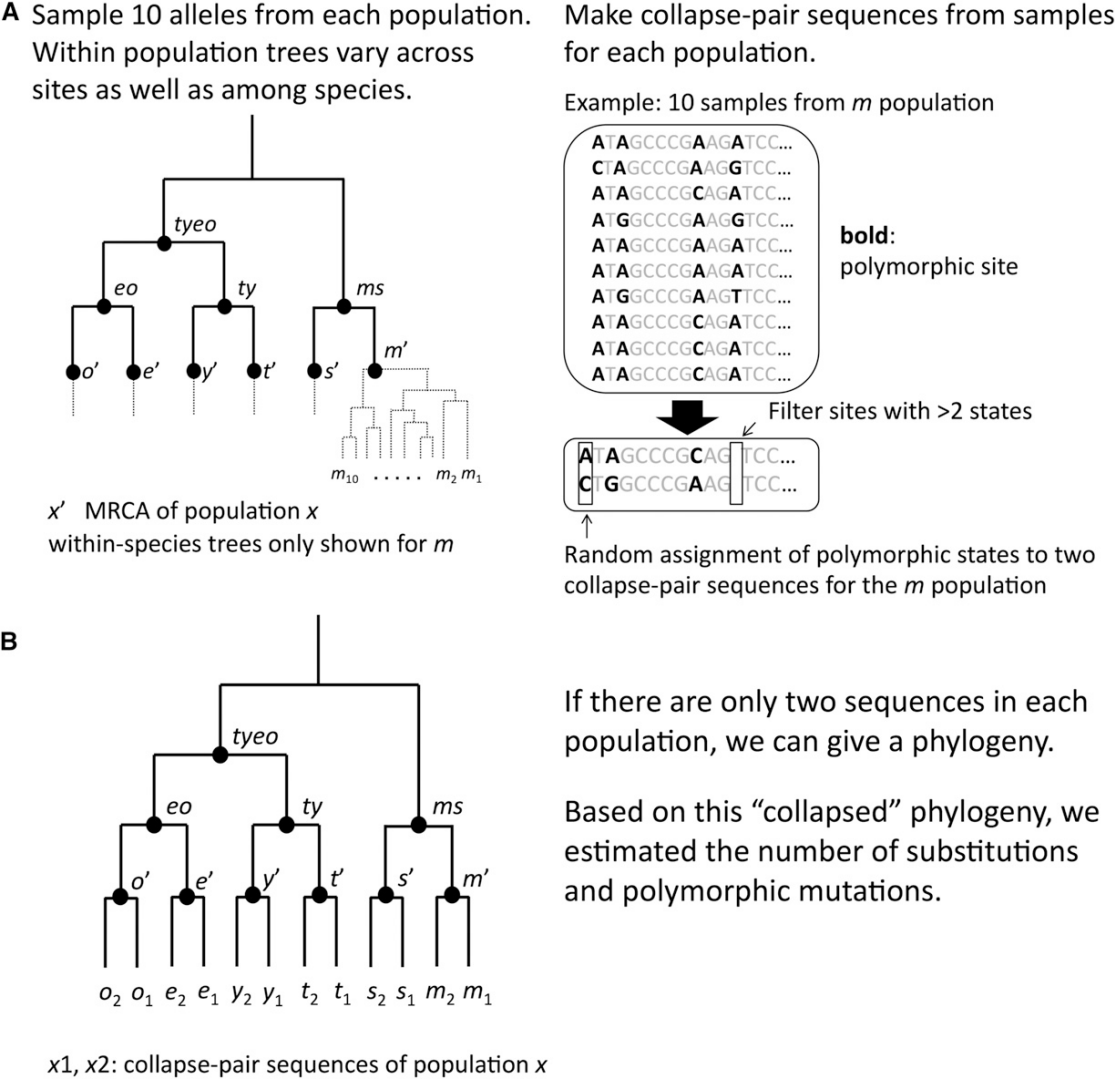
Process for creating input data for bifurcating tree (BT) ancestral inference. (A) The process to make two “collapse-pair” sequences in the BT method. (B) phylogeny used in the BT method. Node names are from Figure 1. *x*’ shows the MRCA of population (species) *x. x*_1_ and *x*_2_ are collapse-pair sequences.

In the BT method, we constructed a pair of representative sequences (referred to as “collapse-pair” sequences) from the population samples for each species. The detailed process is shown in Figure 2. For sites that are monomorphic within the species sample, the same nucleotide was assigned to both collapse-pair sequences. At polymorphic sites, the two segregating nucleotides were assigned randomly to the collapse-pair sequences. Note that site frequency information is lost and associations among sites are randomized in this approach. The phylogeny shown in Figure 2B was assigned to 2×6 collapse-pair sequences and we inferred probabilities of ancestral states at the nodes representing the common ancestors of the collapse pair sequences (*e.g.*, *m*’, *s*’, *t*’, *y*’, *e*’ and *o*’). In the BT and other methods below, we compared the inferred ancestral states at the *X*’ node with observed states among the 10 sampled sequences to estimate the numbers of polymorphic mutations and their SFS in the *a* population. We estimated the numbers of fixations on the *ab-a*’ lineage using the EMC approach.

### Ancestral inference method 3: Star tree (ST)

For ST inference, we assigned a star phylogeny (a single root node for within-species data) to each sample of 10 sequences for ancestral inference. To reduce computational time, we applied this method only to the *m* population. For each of the other five terminal nodes (species), we employed a single sequence consisting of the ancestral nucleotide states of each site (*s*’, *t*’, *y*’, *e*’ and *o*’ nodes in Figure S1). Note that this effectively reduces terminal lineage branch lengths compared to input data used for the other inference methods. Using the phylogeny shown in Figure S1, we inferred ancestral states at node *m*’ and estimated the numbers and SFS of polymorphic mutations. We also estimated the numbers of fixations on the *ab-a*’ lineage using the EMC approach.

### Ancestral inference method 4: Bifurcating tree with weighting (BTW)

The BT method does not incorporate allele frequency information within the sampled nucleotide sequences and we expected inference error and/or biases to arise from effectively assuming that all site frequencies are 0.5. We employed a straightforward approach to weight probabilities of ancestral configurations using an expected SFS. We refer to this approach as the BTW method. In most cases, we used an expected SFS for neutral mutations sampled randomly from a population at equilibrium (SFS_ne_, Fisher 1930; Wright 1969) for weighting. Consider a site configuration with *i* nucleotides in state *A*_1_ and *n* - *i* nucleotides in state *A*_2_. The BT approach gives ancestral probabilities *P*_BT_(*A*_1_) and *P*_BT_(*A*_2_) for ancestral states *A*_1_ and *A*_2_, respectively, at this site. We weight these probabilities using the SFS_ne_. We calculated *F*(*i*) and *F*(*n* - *i*), the expected proportions of polymorphic sites with derived states with counts of *i* and *n* - *i* in a sample of *n* sequences respectively using equation [30] in Sethupathy and Hannenhalli (2008). We calculated the expected proportion of sites where nucleotide *A*_1_ is the ancestral state, *P_ne_*(*A*_1_) = *F*(*i*) / [*F*(*i*) + *F*(*n* - *i*)] and the expected proportion of sites where nucleotide *A*_2_ is the ancestral state *P_ne_* (*A*_2_) = *F*(*n* - *i*) / [*F*(*i*) + *F*(*n* - *i*)]. The weighted joint probabilities of ancestral configurations are *P_BTW_*(*A*_1_) = *P*_BT_(*A*_1_) *P_ne_*(*A*_1_) and *P_BTW_*(*A*_2_) = *P*_BT_(*A*_2_) *P_ne_*(*A*_2_). If the estimated ancestral state differs from both segregating nucleotides, we set the probability (*P_BTW_*) to zero (note that such sites were very rare and showed low probabilities in the bifurcated tree method, in most case ≈ 0.01). We then adjusted joint probabilities so that, at each site, the probabilities of possible ancestral configurations sum to one. We refer to the BTW method using SFS_ne_ as BTW_ne_.

We also examined an iterative approach to SFS estimation. An initial round of ancestral state/SFS inference is conducted under BTW with an arbitrary SFS for weighting (*e.g.*, BTW_ne_ using SFS_ne_). The resulting SFS is employed for weighting in the next round of BTW analyses and this process is repeated. We refer to this method as iterative BTW_est_. Note that we used the AWP approach to estimate the number of fixations because the BTW methods adjusts joint probabilities at ancestral states by SFS weighting and the EMC approach is not applicable.

### Data availability

Computer programs and input files used to simulate sequence evolution, generate collapse-pair sequences and conduct BTW analysis are available at https://github.com/tomotakamatsumoto/Matsumoto_Akashi_AIpoly_codes. Open source license information is available at https://opensource.org/licenses/MIT. Figure S1 shows the phylogeny used in the ST method. Figures S4 and S7 show the actual SFS’s for the simulation scenarios. Table S14 shows the comparison of the SFSs estimated under BTW and the method proposed in Keightley *et al.* (2016). Other supplementary figures and tables show the accuracy of fixation and polymorphism inference under several evolutionary scenarios and methods. All supplementary figures and tables are provided as a single file. Supplemental material available at Figshare: https://doi.org/10.25387/g3.5996342.

## Results

### Stationary scenarios

We first examined the performance of three previously suggested ancestral inference methods that have been employed to estimate the numbers polymorphic mutations and their SFS and the numbers of fixations among closely related species. We discuss results for stationary base composition scenarios without selection (GC_0_ = 0.5) and with selection (GC_0_ = 0.7) for the bifurcating tree (BT) and star tree (ST) methods because these approaches showed contrasting biases in the estimation of SFS. We then compare results for the single allele (SA) method.

**Figure 3:**
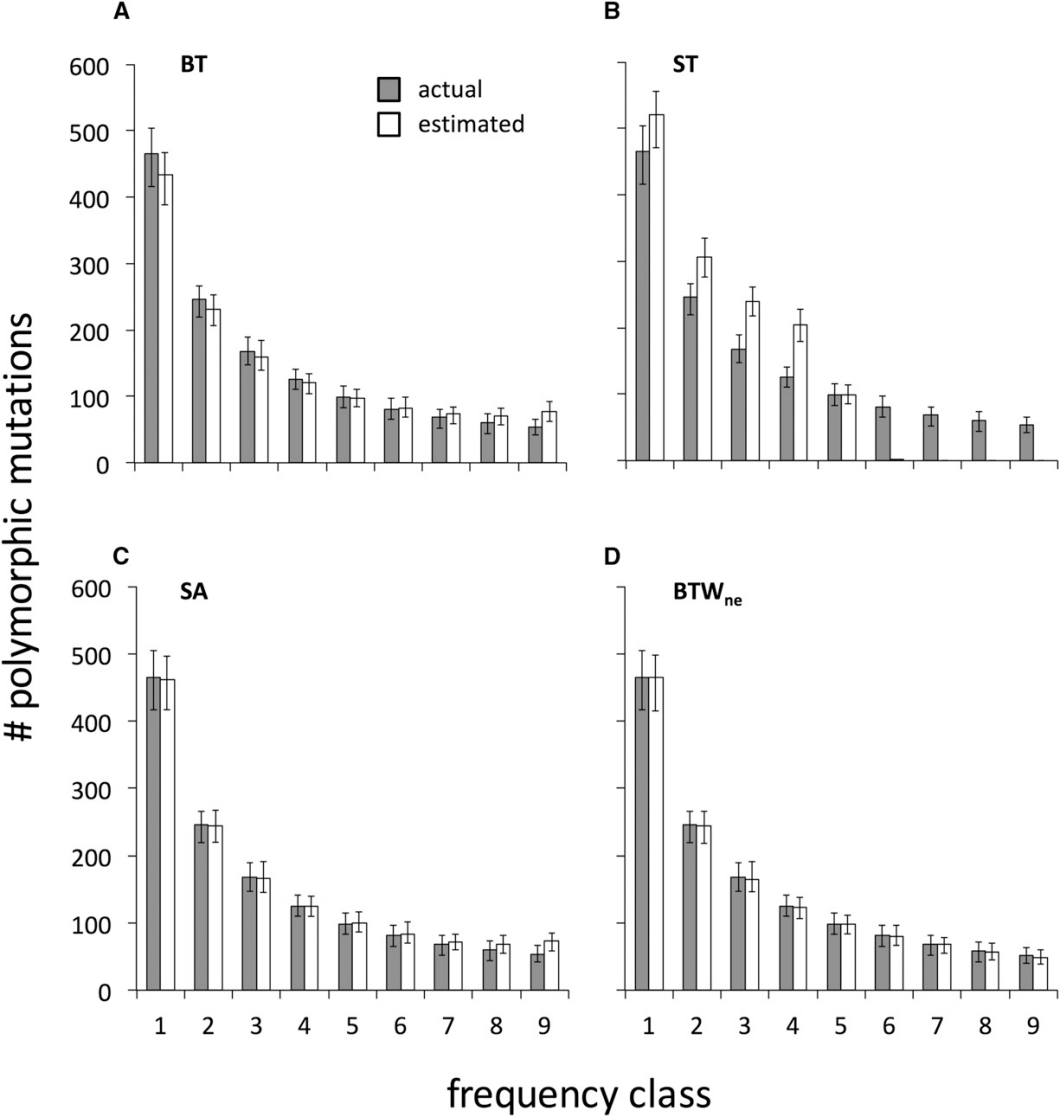
Actual *vs.* estimated numbers of polymorphic *pu* mutations for each frequency class. Results for four inference methods: BT, ST, SA and BTW_ne_ are shown. The legends applies across graphs. The simulation assumed stationary evolution with GC_0_ = 0.5 and results are shown for the *m* population. Population sampling and following ancestral inference were replicated 100 times. The figures show the average and the 95% confidence interval among the replicates. The scales of unlabeled axes are shared across graphs in the same columns and in the same rows. This standard applies to all following figures.

**Figure 4:**
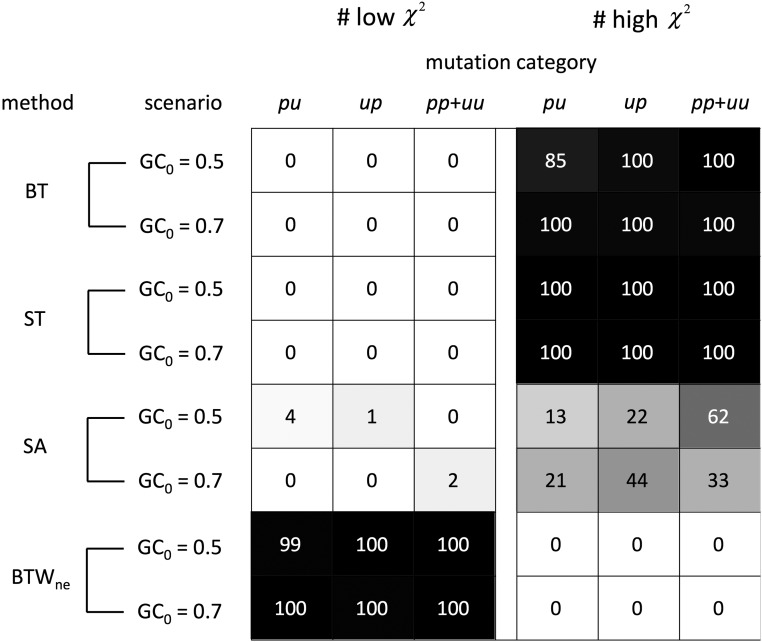
Performance of ancestral inference methods for estimating the SFS of polymorphic mutations in stationary GC content scenarios. $\chi^{2}$goodness of fit statistics were calculated using actual *vs.* estimated numbers of polymorphic mutations for each mutation category. In each frequency class, the proportions of actual polymorphic mutations were used to calculate “expected” values to compare to the inferred numbers of polymorphisms (“observed” values). $\chi^{2}$ statistics were calculated for each replicate with these expected and observed values. The gray scales and cell values give the numbers of replicates showing “poor” fits between observed and expected values ($\chi^{2}$ ≥ 13.0) and “good” fits ($\chi^{2}$ ≤ 3.5). Low and high $\chi^{2}$ cutoffs correspond to *P* ≥ 0.9 and ≤ 0.1 for $\chi^{2}$ goodness of fit tests with the degree of freedom = 8. Note that $\chi^{2}$ values strongly depend on the number of polymorphic mutations; results are comparable among different methods for the same simulation scenario and mutation category, but are only comparable among different scenarios or mutation categories if their sample sizes are similar. The numbers of polymorphic mutations in each scenario and mutation category are shown in Table S1. The SFS of the *m* population was estimated under four inference methods: BT, ST, SA and BTW_ne_. The simulation assumed stationary evolution with GC_0_ = 0.5 and 0.7. Population sampling and ancestral inference were replicated 100 times.

The BT method biases the estimated SFS toward a “flat” (uniform) distribution. For the *m* population in GC_0_ = 0.5, this bias causes underestimation and overestimation of the proportions of polymorphic mutations in low and high frequency classes, respectively (Figure 3A). Such errors bias SFS toward patterns consistent with positive selection or deviation from equilibrium after population size reduction. We measured goodness of fit between estimated and actual SFS to compare the estimation accuracy among methods (Figure 4, Table S1). Accuracy of the estimation of the total numbers of polymorphic mutations was examined separately (Figure S2). BT inference results in substantial differences between estimated and actual SFS for all mutation categories both at GC_0_ = 0.5 and GC_0_ = 0.7.

BT estimation employs collapse-pair sequences that are constructed without regard to the prevalence of the states in the within-species sample. This procedure biases ancestral inference toward equal probabilities that each polymorphic state is ancestral. Importantly, in GC_0_ = 0.7, estimation error is greater for *up* than for *pu* mutations and results in a stronger bias toward a flat SFS for *up* changes (Figure S3A and B). Such patterns arise because the numbers of polymorphisms differ among mutation categories. In GC_0_ = 0.7, *pu* mutations outnumber *up* because of high GC content and therefore, misidentifying *pu* as *up* has a larger proportionate impact on estimated *up* counts.

Ancestral inference errors can bias tests of natural selection. Differences in the SFS between different mutation categories interspersed within DNA can reveal differences in their fitness effects (Sawyer *et al.* 1987). In GC_0_ = 0.5, all mutations evolve neutrally and their SFS are similarly biased under the BT method. However, in GC_0_ = 0.7, the stronger bias toward a flat SFS for *up* mutations than for *pu* mutations can exaggerate differences in their SFS. In the stronger selection case, this bias increases because the ratio of *pu* to *up* polymorphisms increases (in GC_0_ = 0.9, the estimated number of *up* mutations in frequency class nine is about twice the actual value). Note that BT biases should not produce false signals of selection if high GC is maintained by mutation biases at equilibrium because the expected numbers of *up* and *pu* polymorphisms do not differ in such a scenario. *Changes* in mutation rates, however, can lead to unequal numbers of polymorphic mutations among mutation categories when base composition has not reached equilibrium. For example, if GC → AT mutation rates increase, the numbers of *pu* mutations will become larger than that of *up*. In such a case, the stronger estimation bias toward a flat SFS for *up* mutations could cause a false positive signature of fixation bias.

Although SFS estimation is unreliable under the BT method, the total numbers of polymorphic mutations appear to be estimated accurately (Figure S2). This similarity between estimated and actual values appears to reflect a counter-balance between overestimation of the number of polymorphic mutations in high frequency classes and underestimation of those in low frequency classes and may not apply for other evolutionary scenarios.

**Figure 5:**
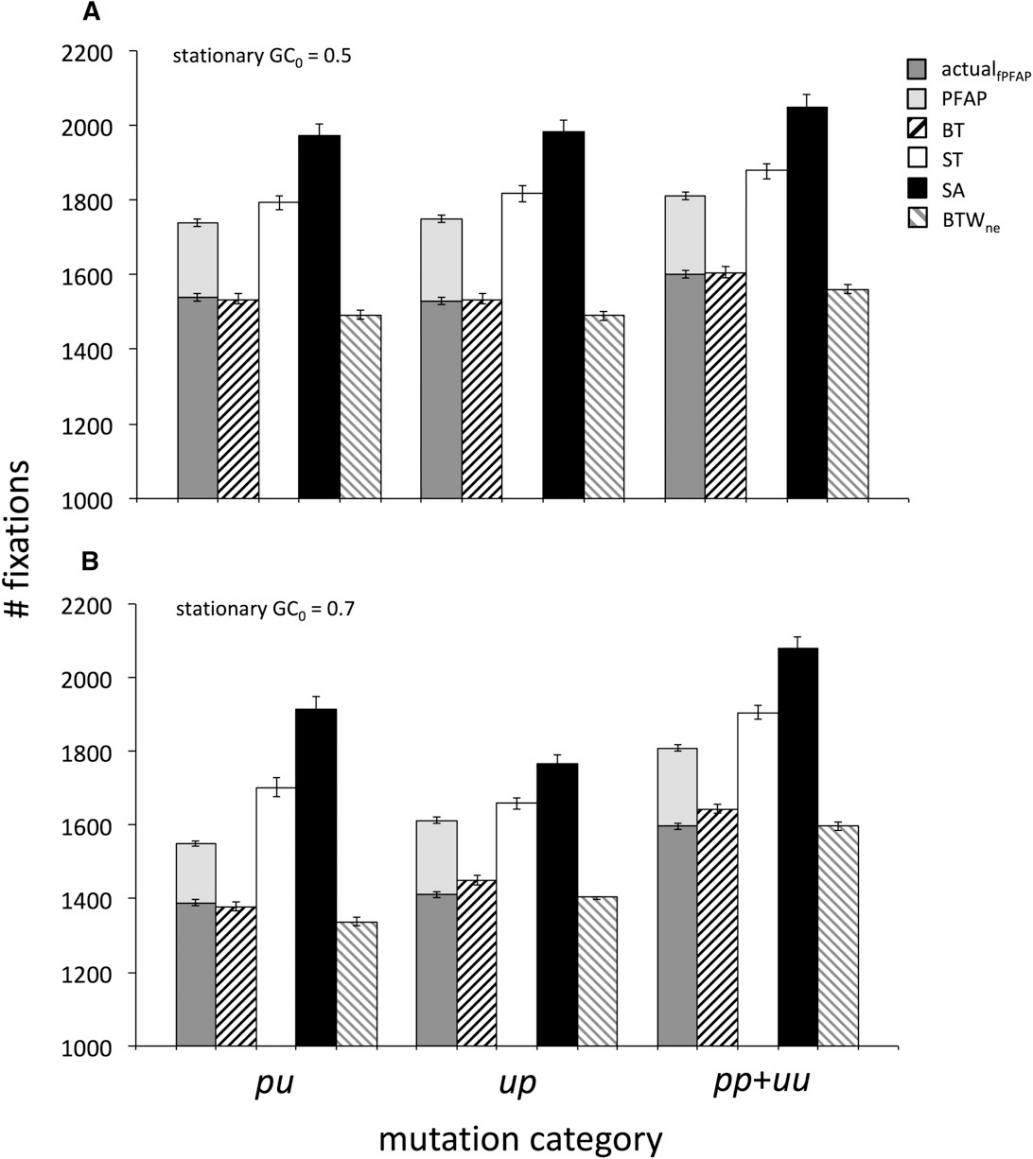
Actual *vs.* estimated numbers of fixations under four ancestral inference methods. Results are for fixations in the *ms-m*’ lineage (*ms-m* lineage in SA). Among actual fixations, parallel fixations of ancestral polymorphism (PFAP) in the *ms-m*’ and *ms-s*’ lineages are shown separately from non-PFAP fixations (actual_fPFAP_). The legend applies to both graphs. The simulation assumed stationary scenario with (A) GC_0_ = 0.5 and (B) 0.7. Population sampling and ancestral inference were replicated 100 times. Averages and 95% confidence interval of counts among the replicates are shown. Note that y-axis values do not start at zero.

The BT method underestimates the numbers of fixations in all mutation categories (Figure 5). This bias appears to reflect a difference between our simulation of population genetic processes and BASEML parameterization caused by parallel fixations of ancestral polymorphism (PFAP). BASEML employs a transition-state model that assumes parameter homogeneity (across sites and over time) within lineages. “Fixations” in our simulations are heterogeneous; for a given transition (ancestral and derived state), the probability of parallel fixations of ancestral polymorphism in sister species is considerably higher than the probability of independent originations followed by fixations in two lineages. We confirm that the estimated number of fixations are similar to actual values filtered for PFAP occurrences (actual_fPFAP_) (Figure 5). In our simulations, PFAP events are common because terminal lineages are relatively short, levels of ancestral variation are high, and population sizes are maintained through species splitting events.

In contrast to the BT method, the star tree approach (ST) leads to overestimation of the proportions of mutations in low frequency classes and underestimation of those in high frequency classes (Figure 3B). Under this method, inference is strongly biased toward attributing the prevalent state in the sample as ancestral because such configurations reduce the required numbers of changes in the tree (Figure S1). For example, consider a site at which the frequencies of two polymorphic nucleotides, A and T, are 0.6 and 0.4, respectively in a sample of 10 sequences. The scenario in which T is ancestral requires six independent changes compared to four changes for the scenario in which A is ancestral. If the probability of nucleotide change within a lineage is low, two additional changes would greatly decrease the probability that the rare state, T, is ancestral. Overestimation of the probabilities that common states are ancestral causes over- and under-estimation of the numbers of polymorphisms in low and high frequency classes, respectively (Figure 3B). Differences between actual and estimated SFSs are considerable; the estimated numbers polymorphic mutations in high frequency classes are close to zero (Figure 3B, Figure 4, Table S1).

ST estimation errors may impact data analyses because mutation categories are not equally affected by the biases. Almost all *pu* (*up*) mutations in high frequency classes are mis-inferred to be *up* (*pu*) mutations in low frequency classes. If the numbers of *pu* polymorphisms are larger than the numbers of *up* polymorphisms, the skew toward rare variants in the SFS will be larger for *up* mutations. Such biases are problematic when the SFSs or average frequencies of *pu* and *up* mutations are compared to test for fixation biases caused by weak selection or biased gene conversion in base composition evolution. In scenarios of non-neutral base composition bias (*e.g.*, GC_0_ = 0.7; Figure S3C and D), the estimation error explained above would underestimate the advantageous effect of *up* mutations (or deleterious effect of *pu* mutations), or the effect of biased gene conversion toward GC. In addition, changes in mutation rate can cause a false signature of selection and biased gene conversion (similar to the BT method). For example, SFS of *pu* mutations can show stronger skews toward rare variants if base composition has not reached equilibrium after a *up* mutation rate increase. This can cause a false signature of a deleterious effect of *pu* mutations (or advantageous effect of *up* mutations), or biased gene conversion toward GC.

ST inference also yields inaccurate estimates of the total numbers of polymorphic mutations when base composition is under selection (Figure S2B). In this method, almost all *pu* (*up*) mutations in high frequency classes are inferred as *up* (*pu*) mutations in low frequency classes. Therefore, the difference in the total numbers of polymorphic mutations in GC_0_ = 0.7 causes large over- and under-estimation of the total numbers of *pu* and *up* polymorphic mutations, respectively (Figure S2B, S3C and D). In the neutral case (GC_0_ = 0.5), the total numbers of *pu* and *up* polymorphic mutations are almost equal and therefore, the counter-balancing of the errors results in similarity of estimated and actual numbers of polymorphic mutations (Figure S2A). This error-balancing also occurs when high GC is maintained by mutation rate bias at equilibrium without selection.

The ST method shows relatively accurate estimation of the numbers of fixations (Figure 5). This accuracy also appears to reflect counter-balancing effects among different errors. The ST method overestimates the numbers of fixations by attributing polymorphic states at higher frequency to the MRCA node. This overestimation can be partly compensated by the underestimation because of PFAP. However, the compensation is not exact and the ST method shows overestimation of the number of fixations when fixation biases affect base composition (Figure 5).

Differences in polymorphism/divergence ratios (*r*_pd_) among mutation categories interspersed within DNA can reveal differences in their fitness effects (McDonald and Kreitman 1991). In the ST method, estimation errors for the total numbers of polymorphic mutations can be considerable and can differ in direction among mutation categories (Figure S2B). For example, for GC_0_ = 0.7, *r*_pd_ for *pu* and *up* mutations are 15% underestimated and 7% overestimated, respectively [this reflects 6% underestimation and 10% overestimation of the numbers of polymorphisms (Figure S2B) and 9% and 3% overestimation of the numbers of fixations for *pu* and *up* mutations, respectively (Figure 5)]. Because mutation-selection-drift predicts higher *r*_pd_ for deleterious *pu* mutations than for advantageous *up* changes (Akashi 1995), these errors can diminish evidence for weak selection/fixation biases in *r*_pd_ comparisons between these mutations categories (*i.e.*, reduce the absolute values of estimated fitness effects). At mutational equilibrium in the absence of fixation biases, the estimation of the numbers of polymorphic mutations is accurate and *r*_pd_ does not differ between *pu* and *up* mutations. However, an increase in *up* mutation rate can cause an elevated ratio of *up* to *pu* polymorphism and generate a signal similar to fixation biases favoring *up* mutations if base composition is on the approach to equilibrium under a new mutation rate.

The SA and BT methods show similar estimation biases toward flat SFS (Figure 3A and C). The SA method is more accurate but deviations from actual values remain appreciable (Figure 4, Table S1). An important source of error in SA is the assumption of identity of states at the within-species MRCA and sibling species nodes (*e.g.*, *m*’ and *ms*). In our neutral simulation, approximately 5.2% of sites underwent fixations in *ms-m*’ lineage. Polymorphic sites in the *m* population that experienced fixations in the *ms-m*’ lineage violate SA inference assumptions. For example, consider a site at which, in a sample of 10 sequences from *m* population, two nucleotides G and T segregate with frequencies 0.9 and 0.1, respectively. If the actual ancestral state at *m*’ is G as a consequence of a T→ G fixation in the *ms-m*’ lineage, SA estimates a T → G polymorphic mutation with frequency 0.9, rather than a G → T mutation with frequency 0.1. In this example, SA would overestimate high frequency *up* polymorphisms and underestimate low frequency *pu* polymorphisms. Estimation error also occurs in the opposite direction (underestimation of low frequency classes and overestimation of high frequency classes). However, if the actual numbers of polymorphic mutations in high frequency classes is much smaller than that in low frequency classes, the overall bias will be toward flatter SFS as shown in Figure 3C. Similar to the BT and ST methods, when the numbers of *pu* and *up* polymorphic mutations differ, the mutation category showing the smaller number experiences stronger inference biases. From these reasons, potential effects of the SA method on SFS comparisons are similar to BT.

The SA method shows slight overestimation of the total number of polymorphic mutations (Figure S2, about 2∼4% of the estimated number of polymorphic mutations are overestimated). If the inferred ancestral state differs from both segregating states, this method “counts” two different polymorphic mutations at such a site. Thus, fixations in the *ms-m*’ lineage can cause overestimation of the total number of polymorphic mutations under SA. Consider a case similar to the example above with G and T segregating at frequencies 0.9 and 0.1, respectively but in which C is assigned as ancestral at the *ms* node. If the actual ancestral state at *m*’ is G as a consequence of a C→ G fixation in the *ms-m*’ lineage, the SA method estimates a C → G polymorphic mutation with frequency 0.9 and a C → T mutation with frequency 0.1, rather than a single G → T mutation with frequency 0.1. The magnitude of overestimation becomes larger for *up* than for *pu* mutations with increasing GC bias because of the reduced ratio of *up*:*pu* polymorphism. In addition, overestimation increases with the numbers of fixations in the lineage prior to the within-species MRCA.

The use of a single allele in the SA approach may lead to error in estimating numbers of fixations. Mutations on terminal lineages fall into two classes: those that occurred prior to and those that occurred after the MRCA of the population sample. The latter class are polymorphic in the sample but are counted as fixations under SA. Thus, the numbers of fixations are overestimated as a function of the depth of the MRCA within the terminal lineage. In GC_0_ = 0.5, roughly 22% of the estimated *pu* and *up* “fixations” under SA are actually polymorphic among the 10 sequences sampled from a population. Filtering all polymorphic sites yields estimates of the numbers of fixed difference under SA similar to actual_fPFAP_ (data not shown). However, such an approach complicates the interpretation of polymorphism/divergence analysis because different sets of nucleotide sites are examined at short *vs.* long time scales.

Under neutral evolution (including GC_0_ = 0.5), *r*_pd_ estimation biases are similar for *pu* and *up* mutations and we do not expect false positives (Figure 5, Figure S2). However, in GC_0_ = 0.7, the average proportions of post-MRCA changes are roughly 27% and 18% for *pu* and *up*, respectively. Differences in the rate of accumulation of mutations on the pre- and post-MRCA parts of the lineage among fitness classes can impact *r*_pd_ comparisons. As shown in Figure 5B, the magnitude of the overestimation of the number of fixations is much higher for *pu* mutations (roughly 23%) than for *up* mutations (roughly 10%). Estimation error is much smaller for the numbers of polymorphic mutations than for fixations (Figure S2). In total, *r*_pd_ values for *pu* and *up* mutations are about 18% and 5% underestimated, respectively, in GC_0_ = 0.7, which diminishes *r*_pd_ differences (and the signal of selection) when fitness effects differ between mutation categories.

SA inference can also bias tests of equilibrium base composition among lineages. Comparisons of the numbers of *pu* and *up* fixations can be employed to infer the direction of base composition evolution among lineages and the causes of non-stationary evolution (Akashi *et al.* 2007). Greater overestimation of the numbers of *pu* fixations than that of *up* causes a false signal of decline of the favored state (excess *pu* fixations). The magnitude of inference error depends on the strength of selection/fixation biases and can produce a pattern similar to expectations under a uniform reduction in fixation biases within a lineage (Akashi *et al.* 2007).

In summary, the three methods examined above can show considerable error in estimating the numbers and SFS of polymorphisms as well as the numbers of fixations among closely related species. The SA method shows the most accurate estimation of SFS among the three methods but can suffer from error in the estimation of the total numbers of polymorphic mutations and fixations. For estimation of the total numbers of polymorphic mutations and fixations, the BT method is the most accurate among these approaches.

The frequencies of polymorphic mutations themselves contain information about the identity of ancestral and derived states (Watterson and Guess 1977; Griffiths and Tavare 1998). Under neutral and weak selection cases, substantially larger proportions of derived mutations are expected to be found at low frequencies within a population (Fisher 1930; Wright 1969). We attempted to incorporate such information to improve SFS estimation under BTW. We began by employing the expected SFS under neutral equilibrium, SFS_ne_, for weighting. Figures 3D, Figure 4 and Table S1 show that this method, BTW_ne_, more accurately estimates SFS than BT, ST, and SA. Estimation remains reliable for some scenarios of weak selection on base composition where the SFS_ne_ assumption is violated (Figure 4, Table S1, Figure S4B). The numbers of polymorphic mutations are also estimated reliably under BTW_ne_ (Figure S2) but the numbers of fixations are underestimated because of PFAP (Figure 5). Fixation numbers are close to actual_fPFAP_ values but are underestimated because the AWP approach does not adjust for multiple fixations (Matsumoto *et al.* 2015).

### Non-stationary scenarios

**Figure 6:**
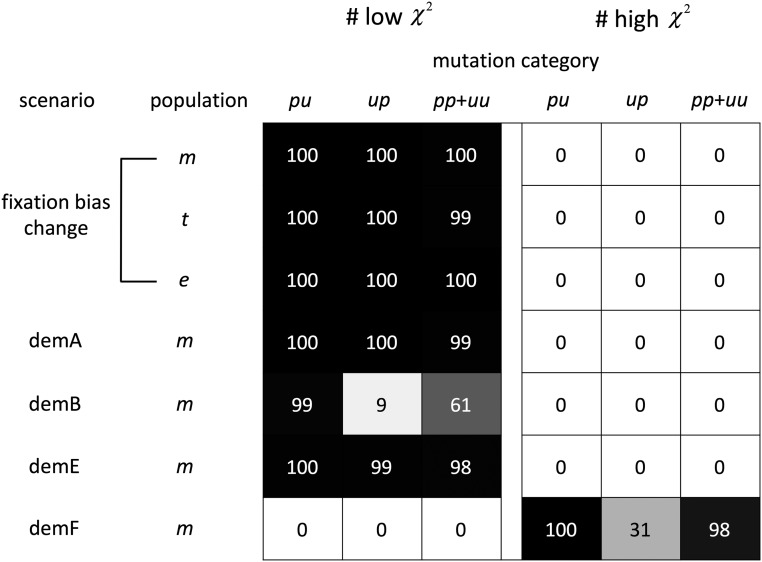
Performance of the BTW_ne_ methods for estimating the SFS of polymorphic mutations in non-stationary GC content scenarios. $\chi^{2}$ goodness of fit statistics were calculated using actual *vs.* estimated numbers of polymorphic mutations for each mutation category. The gray scale and cell values give the numbers of replicates showing “poor” and “good” fits between observed and expected values. The procedure of $\chi^{2}$ calculation and the meaning of the gray scale and the number inside each cell are described in the Figure 4 legend. The numbers of polymorphic mutations in each scenario and mutation category is shown in Table S2. The SFS of the focused population was estimated under the BTW_ne_ method. The simulation assumed fixation bias change scenario with GC_0_ = 0.7 and four demographic change scenarios, demA, demB, demE and demF. Population sampling and ancestral inference were replicated 100 times.

We examined the robustness of the BTW_ne_ method in non-neutral, non-stationary scenarios of base composition evolution. In the fixation bias change scenario with GC_0_ = 0.7, the BTW_ne_ method accurately estimates the SFS (Figure 6, Table S2) and numbers of polymorphic mutations (Figure S5). These results suggest robustness of weighting by SFS_ne_ in at least some scenarios in which *pu* and *up* mutations are not neutral and base composition is not stationary. Similar to the results for stationary scenarios, BT, ST and SA methods did not show the accurate estimation of SFS (Table S3). We also found that the numbers of fixations show PFAP and AWP underestimation under BTW_ne_ (Figure S6), which is also consistent with the results for stationary scenarios.

To test the robustness of the BTW method to demographic violations of the standard neutral model, we analyzed sequences simulated under four demographic change scenarios. These scenarios result in substantial departures from SFS_ne_ (Figure S7); demA and B show a flatter SFS whereas demE and F show excesses of rare mutations. Results for two other scenarios (demC and D) that showed smaller departures from SFS_ne_ are given in the supplementary materials (Figure S7; Table S2).

BTW_ne_ reliability varied considerably among the tested demographic scenarios: SFS estimation was accurate for demA and demE but was unreliable for demB and demF (Figure 6, Table S2). However, estimation of the total numbers of polymorphic mutations is accurate for all four scenarios (Figure S8). The estimated numbers of fixations in the *ms-m*’ lineage under BTW_ne_ is also very close to actual_fPFAP_ (Figure S9).

We tested whether ancestral inference error for demB and demF can be attributed mainly to incorrect SFS weighting. If this is the case, then employing an accurate SFS for weighting (without changing any aspects of the upstream ancestral state inference) should improve estimation accuracy. Table S4 shows marked increases in the accuracy of SFS estimation for demB and demF when the average actual SFS’s among replicates is employed as the “empirical” SFS (BTW_emp_).

### Strongly biased base composition scenarios

In stronger selection (or fixation bias) scenarios, actual SFS show strong departures from SFS_ne_ (Figure S4C, E and F). BTW_ne_ can accurately estimate the SFS in some of these scenarios (Table S5). The total numbers of polymorphic mutations are accurately estimated (Figure S10), and fixations are similar to actual_fPFAP_ (Figure S11) in all of the considered scenarios. However, similar to the demographic change scenarios, strong departures from SFS_ne_ can result in considerable reductions in the accuracy of SFS estimation under BTW_ne_ (*e.g.*, *ty-t* lineage in the fixation bias change scenario in Table S5). Again, incorrect SFS weighting appears to be the main issue; BTW_emp_ gives accurate SFS estimation for the strong and/or non-equilibrium base composition bias scenarios (Table S4).

### Iterative BTW_est_

**Figure 7:**
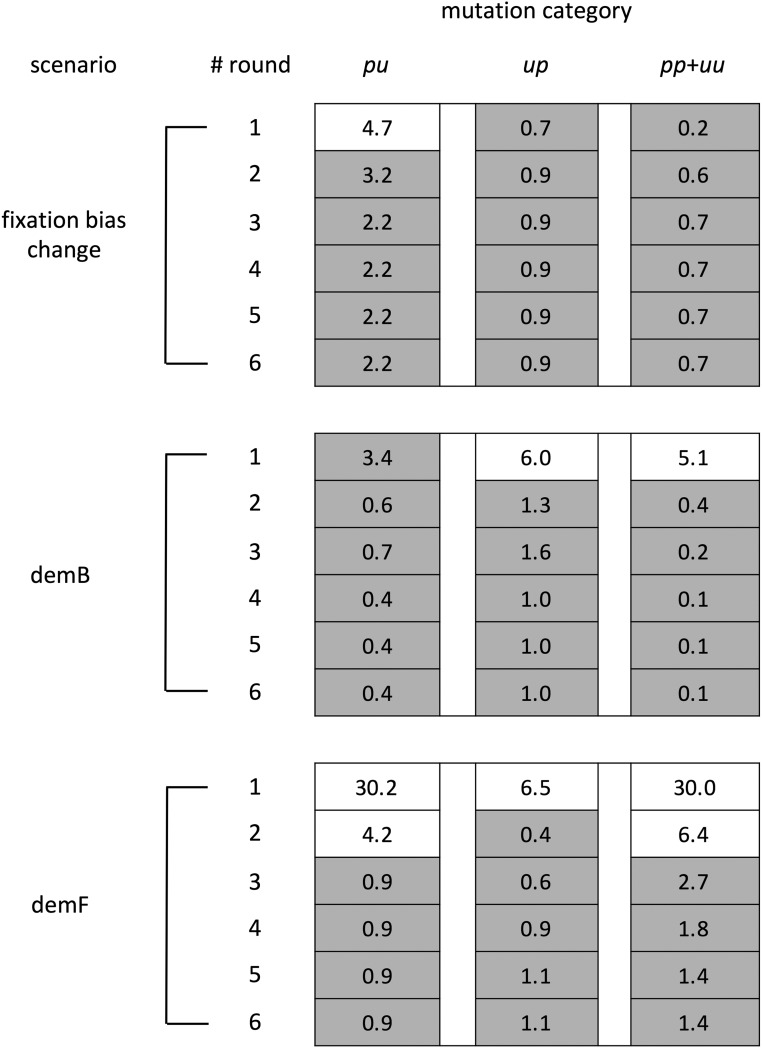
Performance of the iterative BTW_est_ method for estimating the SFS of polymorphic mutations in non-stationary GC content scenarios. SFS of the *t* population in fixation bias change scenario with GC_0_ = 0.9 and *m* population in demB and demF were estimated under the iterative BTW_est_ method, and $\chi^{2}$ goodness of fit statistics were calculated using actual *vs.* estimated numbers of polymorphic mutations for each mutation category. The procedure for $\chi^{2}$ calculation is described in the Figure 4 legend. This figure shows results for a single replicate that showed relatively large $\chi^{2}$ value in the BTW_ne_ analysis. The estimation under iterative BTW_est_ was repeated for six rounds (the first round was BTW_ne_ and the following five were BTW_est_ using the estimated SFS of the previous round). The cell values show the calculated $\chi^{2}$ value and the shaded cell means that the $\chi^{2}$ ≤ 3.5 which is the criteria of low $\chi^{2}$ value. The results of all 100 replicates for demB and demF are shown in Table S5.

The BTW_ne_ method shows robustness to moderate differences between the assumed and actual SFS but stronger departures can degrade the accuracy of ancestral inference. Because weighting by realistic SFS (BTW_emp_) can yield accurate inference in such cases, iterative SFS estimation may be a promising approach for ancestral inference when SFS_ne_ may be strongly violated. To test this notion, we performed ancestral inference followed by multiple rounds of SFS weighting. The first round employed SFS_ne_ for weighting and the resulting SFS will be referred to as SFS_est1_. The latter was employed to weight a second round of ancestral inference and this process was repeated for five rounds (*i.e.*, the last round employed SFS_est4_ for weighting). Iterative weighting was tested with data from three scenarios, demB, demF and fixation bias change scenario with GC_0_ = 0.9. From each scenario, we choose a single replicate that shows a large departure between actual and estimated SFS under BTW_ne_. Iterative BTW_est_ allows accurate estimation of the numbers of polymorphic mutations and SFS across mutation categories in all of the examined scenarios and appears to provide a feasible method to considerably enhance the accuracy of ancestral inference (Figure 7). In the cases examined, the fit between actual and estimated SFS improved considerably in the second round of estimation (using BTW_est1_) and largely converged within a few rounds (Figure 7). For dem B and demF, we also applied the iterative BTW_est_ method for replicates and confirmed a large improvement in the accuracy of SFS estimation compared with BTW_ne_ inference (Table S2 and S6).

Although the frequencies of polymorphism are informative for ancestral state inference, assuming a particular SFS may bias reconstructions. We tested how the shape of the starting SFS (assumed SFS in the first round) affects the reliability of iterative BTW_est_. We employed SFS_ne_, uniform frequencies and excess common SFS_ec_ (SFS_ne_ was flipped on the vertical axis) as starting SFS for three independent iterative BTW_est_ processes for data from the stationary scenario with GC_0_ = 0.5. SFS_ne_ as the starting SFS is a realistic assumption in this case, but the other starting SFS differ considerably from the actual SFS. The iterative BTW_est_ analysis accurately estimated the SFS with all three starting SFS (Table S7). Estimated SFS under iterative BTW_est_ can converge to the accurate values despite inaccuracies in the assumed SFS in the first round (see also Table S6).

Our main simulation scenarios did not included biased mutation or genetic linkage but we show reliable SFS estimation for *pu*, *up* and *pp*+*uu* mutations in such cases (Table S8 and S9).

### Ancestral polymorphism

As we noted above in the discussion of PFAP, shared ancestral polymorphism violates the assumptions of the TRMFT approach and can lead to bias/error in ancestral inference. At least three cases can be problematic: 1) parallel fixations of non-ancestral states (PFAP), 2) maintenance of ancestral polymorphism among sister lineages, and 3) fixation of the non-ancestral state in one lineage and maintenance of both states as polymorphism in the other. Cases 2 and 3 affect polymorphism analysis but in our simulations, polymorphic mutations that originated in an ancestral population and remain shared in sister species are rare (*e.g.*, about 1% of all polymorphic mutations are shared between the *m* and *s* populations in the stationary scenario with GC_0_ = 0.5). To test the effect of shared ancestral polymorphism on the accuracy of ancestral inference (the input tree is incorrect for such sites), we examined the performance of the BTW_ne_ method for data simulated under a tree with shorter branch lengths (2,000, 1,000 and 100 generations) for the *ms-m* and *ms-s* lineages (all other branch lengths are unchanged). These branch lengths result in about 5%, 10% and 25% shared polymorphism, respectively. Among these scenarios, the reliability of BTW_ne_ inference is high at 5 and 10% shared polymorphism but decreased at 25% (Table S10). Importantly, filtering sites showing the same polymorphic states in the *m* and *s* populations (*i.e.*, candidates for shared ancestral polymorphism) does not increase the estimation reliability (Table S11) perhaps because the 3^rd^ class of errors remain unfiltered.

### Ancestral inference under maximum parsimony

We showed that previously employed ancestral inference approaches for polymorphism data, BT, ST and SA, can yield significant estimation error for the numbers of polymorphic mutations and fixations as well as the SFS of segregating sites (Figures 3-5, Figure S2, Table S1, S3). Maximum parsimony (MP) was not examined in detail here because the approach is known to suffer from large estimation error in scenarios with unequal nucleotide transition rates (Collins *et al.* 1994; Perna and Kocher 1995; Eyre-Walker 1998; Galtier and Boursot 2000; Alvarez-Valin *et al.* 2004; Akashi *et al.* 2007; Hernandez *et al.* 2007; Matsumoto *et al.* 2015). We emulated maximum parsimony by combining a Jukes-Cantor transition rate matrix (Jukes and Cantor 1969) and single best reconstruction (Matsumoto *et al.* 2015) and inferred polymorphisms and fixations in the stationary scenario with GC_0_ = 0.7 under MP. As expected, MP shows considerable error in estimating the numbers of fixations at moderate base composition bias even among closely related lineages (Figure S12A) and we believe the approach should not be employed as a sole or primary ancestral inference method (Matsumoto *et al.* 2015). Because the underestimation is much larger for advantageous (*up*) than deleterious (*pu*) mutations, this bias causes a false signal of non-stationary base composition evolution (Matsumoto *et al.* 2015). For SFS estimation, maximum parsimony results in underestimation and overestimation of low and high frequency classes, respectively. The biases are similar in direction, but somewhat lower in magnitude, than for BT inference (Table S12). The numbers of polymorphic mutations are estimated accurately (Figure S12B). *r*_pd_ is 23% overestimated for *pu* mutations and 41% overestimated for *up* mutations. Because *r*_pd_ decreases monotonically with *N*_e_*s*, *r*_pd_ tests will underestimate the advantageous effect of *up* mutations and overestimate the deleterious effects of *pu* changes. In summary, for comparisons of *pu* and *up* mutations, maximum parsimony exhibits moderate bias in the SFS estimation but considerable biases in estimating the number of fixations. The latter biases are expected to strongly impact data analyses.

### Sample size and SFS estimation

Finally, we show how sequence length (and the number of polymorphic sites) affects the reliability of the inference of polymorphisms. In the above analysis, we used 100,000 nucleotide sites. Among these nucleotide sites, about 4,000 are polymorphic among 10 sequences sampled from *m* population in GC_0_ = 0.5. We also conducted analyses using subsets of this data (10,000 and 1,000 sites) for the stationary scenario with GC_0_ = 0.5. We analyzed 10,000 and 1,000 sites from the same sequences used in the above analysis and estimated the SFS under the BTW_ne_ with 100 replications. Our result shows that even for the smaller data set, the estimated SFS is quite accurate. With 10,000 sites, all 100 replicates showed close matching between estimated and actual SFS for *pu*, *up* and *pp*+*uu* mutations. With 1,000 sites, the very small numbers of polymorphic mutations precluded goodness of fit evaluation but the estimated SFS’s are close to actual (Table S13). However, we have to note that the accurate estimation of SFS with 1,000 sites may not be a general result. Complexity of the evolutionary scenario or the number of analyzed species can affect the number of sites required for the accurate estimation. In addition, if bootstrap analysis is conducted to calculate the confidence interval of SFS, ancestral inference with small number of sites can show highly variable result across replicates (Matsumoto *et al.* 2015) which makes the confidence interval large.

## Discussion

The rapid expansion of genome-scale polymorphism data has generated a need for methods to infer ancestral and derived states for within-species variation. Numerous studies have demonstrated the impact of nucleotide transition model on the accuracy of TRMFT-based ancestral inference (*e.g.*, Collins *et al.* 1994, Perna and Kocher 1995, Eyre-Walker 1998, Galtier and Boursot 2000, Alvarez-Valin *et al.* 2004, Akashi *et al.* 2007, Matsumoto *et al.* 2015). However, for genomes undergoing recombination, small genetic regions, even individual sites, can have different genealogies in within-species samples. In such cases, the requirement for a single phylogeny is an important limitation of likelihood-based approaches to ancestral inference. Because region-specific gene trees may be difficult to reconstruct accurately (especially when the regions are small), we have explored approximate approaches to infer fixations and polymorphisms among closely related lineages.

Several previous studies have employed likelihood-based estimation methods using ancestral inference that incorporate population genetic and phylogenetic models to estimate the number of fixations and polymorphic mutations and their SFS. However, most such studies have not employed models (*i.e.*, transition rate matrices) that can incorporate mutation rate biases and differences in fixation probabilities of mutations caused by selection or biased gene conversion. For example, the method proposed in Wilson *et al.* (2011) employs both population genetics theory (expected stationary SFS) and phylogenetic ancestral inference based on a transition rate matrix to calculate likelihoods of states at ancestral nodes. Their main focus was detection of adaptive protein evolution and fixation biases are considered only for non-synonymous changes; synonymous changes are assumed to evolve under the HKY85 model (Hasegawa *et al.* 1985) which has limited applicability for base composition evolution scenarios including those explored here (Matsumoto *et al.* 2015).

More recently, Keightley *et al.* (2016) developed a method to estimate the unfolded SFS. Their method estimates the expected number of nucleotide fixations per site, *K*, that can explain the observed polymorphism in a focal species. The estimated *K* is used to estimate the likelihood of ancestral states at each site. The method can assign different *K* for transition and transversion mutations. However, fitness effects/fixation biases for different mutation categories (*e.g.*, *up*, *pu* and *pp*+*uu*) are not considered and the number of outgroups for the analysis is limited to two. Therefore, this method may also be limited for studying base composition evolution. Variation in fixation probabilities across different mutation categories and non-stationary evolution among multiple lineages are known aspects of synonymous site evolution, and we have focused on ancestral reconstruction approaches that can account for such phenomena. The Keightley *et al.* (2016) method estimates SFS for pooled mutations (pooled SFS) and we compared it with the estimates under BTW. Table S14 shows that both methods estimate the pooled SFS with high accuracy in several evolutionary scenarios. However, in four of five scenarios we considered, BTW showed slightly, but significantly, more accurate estimation than the Keightley *et al.* (2016) method.

The three approaches used in previous studies (ST, SA, and BT) can employ nucleotide transition models that incorporate fixation biases and non-homogeneous evolution (*e.g.*, the GTR-NH_b_). However, our results show that these implementations do not allow reliable inference of fixations and polymorphisms among closely related species. In the BT method, the estimation of the total numbers of polymorphic mutations is accurate but that of the SFS is biased toward flat distributions. The ST method showed the largest error among the three methods. The estimated SFS showed strong under- and over-estimation of the number of mutations in high and low frequency class, respectively. In addition, the estimated total number of polymorphic mutations was also inaccurate. The SA method showed the smallest error in the estimation of the SFS among the three methods. However, this method overestimates the number of mutations in high frequency class and showed large estimation error for the total numbers of polymorphic mutations and fixations. The BTW method proposed here allows accurate inference of polymorphisms under a range of scenarios of base composition evolution. The iterative BTW_est_ approach is more broadly applicable and gave accurate estimation in all scenarios examined in this study including cases where actual SFS showed large departures from SFS_ne_.

In contrast to the inference of polymorphisms, fixation estimation shows error even under BTW. This is because the ancestral inference methods using nucleotide transition rate matrices do not consider parallel fixations of ancestral polymorphism (PFAP). The expected extent of PFAP under neutral evolution can be calculated given information about mutation rate and ancestral population size and the effect on particular data sets must be examined on a case by case basis. Inference errors for fixations and polymorphisms in the previous three methods and in BTW may have critical impacts on inferences of selection and biased gene conversion. We have found that the magnitude and direction of biases depend on the method as well as actual evolutionary scenarios. The possibility that inference biases can produce patterns that mimic biological scenarios of interest (such as relationships between apparent strength of fixation biases and GC content) should be a carefully considered in data analyses.

### Recommendation for data analysis

Our simulation results suggest that BTW and iterative BTW_est_ methods can provide reliable inference of polymorphisms across a range of evolutionary scenarios. For data analysis, data pooling is an important aspect that requires balancing sample size and fit to model assumptions. Larger sample sizes are necessary when employing complex substitution models but the methods assume homogeneity (*i.e.*, shared parameter values) across sites. In many cases, data partitioning by GC content, genomic location (*e.g.*, autosome *vs.* sex chromosomes, euchromatin *vs.* heterochromatin), expression level, etc may be warranted and robustness of results to the binning/pooling strategy should be tested.

BT approaches assume no shared polymorphisms among species but the approach shows some robustness to this assumption. High proportions of shared polymorphisms decrease the accuracy of the estimation of SFS under the BTW method, however (Table S7) and samples should be screened for shared polymorphism before applying these approaches.

Overall, the iterative BTW_est_ method should have broad applicability and is our recommended approach. Although the demographic change scenarios considered above are relatively simple, iterative BTW_est_ converged to accurate estimations within a small number of rounds in all the scenarios we considered. Because the computational cost is small, we recommend 5∼10 rounds of iteration for data analysis. In general, employing information from allele frequencies in ancestral state inference should allow more reliable inference if the assumed SFS is correct. However, if the assumed SFS differs from the actual pattern, then this approach could, in principle, lead to estimation biases. Iterative BTW_est_ allows a test of such biases; for a given data set, iterative SFS estimation can be conducted with a range of starting SFS (uniform, equilibrium neutral, excess rare, etc). The approaches should converge to a similar SFS if the inference is accurate. In this study, we found this to be the case for a range of actual SFS (Table S5, S6). These results support that iterative BTW_est_ is a reliable and robust method that allows accurate estimation of a wide range of SFS.

### Future improvement of ancestral inference for polymorphism data

Our findings emphasize that ancestral reconstruction methods must be considered carefully when analyzing within and between species genetic variation even among closely related species. Prevalent biases in some ancestral reconstruction approaches that can give results that mimic biological scenarios of interest are concerning but the approximate approach, BTW, appears to reliably infer polymorphism and SFS data under a broad range of conditions. However, we note that all of the scenarios that we simulated were at least roughly consistent with the input tree and nucleotide transition model employed for ancestral inference (a GTR model with lineage-specific base composition and rate parameters). Considerable error in inferring polymorphism and fixation patterns can occur when assumptions of the TRMFT approach are violated (especially effects of ancestral polymorphism). Further development of population genetic approaches for ancestral inference may be necessary to overcome such issues.
